# Development of a recombinase polymerase amplification-based chromogenic assay for rapid detection of *Salmonella*

**DOI:** 10.3389/fmicb.2026.1819643

**Published:** 2026-04-10

**Authors:** Chang Liu, Han Ren, Yi Li, Boyang Li, Qiuyu Wei, Xiangdong Xu, Huan Ren

**Affiliations:** 1 School of Public Health, Hebei Medical University, Shijiazhuang, China; 2 Chongqing Key Laboratory of Prevention and Treatment for Occupational Diseases and Poisoning, Chongqing Prevention and Treatment Center for Occupational Diseases, Chongqing, China; 3 Chongqing Municipal Health Commission Key Laboratory for Emergency Poisoning Detection and Acute Care, The First Affiliated Hospital of Chongqing Medical and Pharmaceutical College, Chongqing, China; 4 Xinle Traditional Chinese Medicine Hospital, Shijiazhuang, China; 5 Hebei Medical University, Shijiazhuang, China; 6 Cangzhou Center for Disease Control and Prevention, Cangzhou, China; 7 Hebei Key Laboratory of Environment and Human Health, Shijiazhuang, China

**Keywords:** DNAzyme, G-quadruplex, rapid detection, recombinase polymerase amplification, *Salmonella* spp.

## Abstract

**Introduction:**

*Salmonella* spp. is a leading global cause of foodborne illnesses and poses a significant threat to public health. Rapid and reliable detection of this pathogen is critical for implementing effective prevention and control measures.

**Methods:**

A label-free, equipment-free detection method was developed by integrating recombinase polymerase amplification (RPA) with G-quadruplex (G4)/hemin DNAzyme catalysis. Tandem cytosine repeat-modified primers targeting the *invA* gene were employed to enable the generation of G4 structures after RPA amplification; the resulting G4 amplicons complex with hemin to form a DNAzyme that catalyzes H_2_O_2_-mediated oxidation of 2,2’-azino-bis(3-ethylbenzothiazoline-6-sulfonic acid) diammonium salt (ABTS), producing a naked-eye-detectable color change.

**Results:**

The entire detection process was completed within 1 hour, with a limit of detection (LOD) as low as 12 CFU/ml. Specificity testing confirmed no cross-reactivity with non-target bacteria, including *Shigella flexneri* (*S. flexneri*), *Enterobacter cloacae* (*E. cloacae*), *Listeria monocytogenes* (*L. monocytogenes*), and *Escherichia coli* (*E. coli*).

**Discussion:**

This accurate, portable, and user-friendly detection platform offers promising applications for *Salmonella* spp. monitoring in food safety surveillance, particularly in resource-limited settings.

## 1 Introduction

*Salmonella* spp., a Gram-negative pathogen, is a leading cause of foodborne diseases worldwide. This highly adaptable microorganism poses a substantial threat to public health, causing a wide spectrum of clinical manifestations ranging from self-limiting gastroenteritis to life-threatening systemic infections such as typhoid fever and septicemia (Sánchez-Vargas et al., 2011; Lu et al., 2025). *Salmonella* species are generally classified into two main groups: typhoidal *Salmonella* species, which cause systemic typhoid and paratyphoid fevers, and nontyphoidal *Salmonella* (NTS) species, which typically cause self-limiting gastroenteritis. Notably, invasive NTS (iNTS) disease is a major cause of mortality in immunocompromised individuals, particularly in sub-Saharan Africa. According to the World Health Organization (WHO), *Salmonella* is a major contributor to diarrheal diseases globally, with an estimated 93.8 million infections and 155,000 deaths annually, and is classified by the WHO as a foodborne pathogen associated with moderate to severe risk (Li et al., 2019; Zhang et al., 2019). The development of a rapid and sensitive detection method is urgently needed for accurate identification of *Salmonella* spp. in food products, as timely diagnosis is critical for implementing appropriate therapeutic interventions and preventive measures to mitigate public health risks and economic losses.

Traditional detection of the foodborne pathogen *Salmonella* spp. predominantly relies on bacterial culture techniques, which remain the gold standard due to their high specificity for pathogen confirmation (Deusenbery et al., 2021; Hussain et al., 2021). However, these conventional methods are associated with several critical limitations that hinder their utility in rapid-response scenarios: prolonged turnaround times (typically 3–7 days), labor-intensive protocols, and reduced sensitivity when applied to complex food matrices (Aboutalebian et al., 2021). In contrast, nucleic acid testing (NAT) technologies have emerged as powerful alternatives, enabling direct detection of pathogen-specific genetic sequences without prior bacterial culturing. Currently, numerous well-established NAT platforms—including polymerase chain reaction (PCR), real-time quantitative PCR (qPCR), multiplex PCR, and reverse transcription PCR—have gained considerable attention in food microbiology due to their improved speed and sensitivity (Chahorm and Prakitchaiwattana, 2018; Jung et al., 2018; Aboutalebian et al., 2021). Nevertheless, the reliance of these PCR-based methods on sophisticated laboratory equipment and skilled personnel restricts their applicability in resource-constrained settings and on-site testing scenarios (Chahorm and Prakitchaiwattana, 2018; Jung et al., 2018; Aboutalebian et al., 2021; Zhao et al., 2021). This technological limitation underscores the pressing need for simplified, portable, and cost-effective diagnostic tools suitable for point-of-need detection of *Salmonella* spp. under resource-limited conditions.

Recombinase polymerase amplification (RPA), an isothermal nucleic acid amplification technology, has emerged as a transformative alternative to traditional PCR, addressing many limitations inherent to thermal cycling-based assays (Zhao et al., 2021; Tan et al., 2022). Operating optimally at 37–42 °C within 10–20 min, RPA enables rapid target amplification without the requirement for thermal cycling, making it particularly well-suited for point-of-care testing (POCT) applications (Pumford et al., 2020; Munawar, 2022). However, conventional RPA still suffers from complex and time-consuming post-analytical procedures, such as agarose gel electrophoresis and imaging, which limits its on-site application. The integration of RPA with lateral flow dipstick (LFD) technology has further expanded its diagnostic utility, facilitating the development of rapid, user-friendly detection systems with excellent sensitivity and specificity for on-site identification of bacterial and viral pathogens (Gao et al., 2020; Ma et al., 2020). Notably, RPA-LFD systems can yield visible results within 5–10 min without specialized instrumentation, rendering them promising molecular diagnostic tools for low-resource environments (Du et al., 2018; Gao et al., 2018). However, a critical limitation of current RPA-LFD assays is their reliance on chemically modified primers (typically labeled with digoxigenin and biotin), which significantly increases assay costs and limits scalability for high-throughput surveillance applications in food safety control. Thus, the development of a primer modification-free, rapid, sensitive, and cost-effective RPA-based detection method for foodborne pathogens remains a critical unmet need.

To address the limitations of existing RPA-based methods (e.g., high cost due to modified primers), we herein developed a novel, low-cost detection platform that combines RPA with G-quadruplex (G4)/hemin DNAzyme catalysis for the rapid and specific identification of *Salmonella* spp. A pair of engineered primers targeting the conserved *invA* gene—a well-recognized and validated marker of *Salmonella* spp.—was designed with tandem cytosine repeats at their 5′ ends. Upon RPA amplification, these cytosine-rich sequences facilitate the synthesis of complementary guanine-rich strands, which fold into G4 structures and subsequently bind hemin to form a G4/hemin DNAzyme complex. This complex exhibits peroxidase-like catalytic activity, catalyzing the H_2_O_2_-mediated oxidation of 2,2′-azino-bis(3-ethylbenzothiazoline-6-sulfonic acid) diammonium salt (ABTS), resulting in a visible color change that directly indicates the presence of *Salmonella* spp. This G4/hemin DNAzyme-based readout not only enables instrument-free, visual detection of RPA amplicons—facilitating rapid testing in resource-limited settings—but also leverages enzymatic catalysis for signal amplification, enhancing detection sensitivity beyond conventional RPA. Importantly, unlike RPA-LFD assays, this proposed RPA-G4/hemin DNAzyme platform requires no primer modification, significantly reducing assay cost. Furthermore, to demonstrate the versatility of this platform, we extended its application to the detection of *Bacillus cereus* (*B. cereus*), another significant foodborne pathogen known for causing emetic and diarrheal syndromes. This expansion validates the potential of the RPA-G4 strategy as a universal tool for monitoring diverse bacterial threats. Collectively, this RPA-G4/hemin platform provides a robust, portable, and field-deployable solution for the timely detection of *Salmonella* spp. in food samples, aiding in the prevention and control of foodborne bacterial infections particularly in low-resource settings, and also offers a new strategy for the rapid detection of other foodborne pathogens.

## 2 Materials and methods

### 2.1 Bacterial strains and DNA extraction

The bacterial strains used in this study included *Salmonella enterica* subspecies enterica serovar Typhimurium (*S*. Typhimurium ATCC 14028), *Shigella flexneri* (*S. flexneri* ATCC 25876), *Escherichia coli* (*E. coli* ATCC 8739), *Enterobacter cloacae* (*E. cloacae* ATCC 23355), *Salmonella* Dublin (*S.* Dublin ATCC 15480), *Salmonella enterica* subspecies *enterica* serovar Enteritidis (*S*. Enteritidis ATCC 13076), *Salmonella Paratyphi*-A (*S*. Paratyphi-A CMCC 50973), *Salmonella Paratyphi*-B (*S*. Paratyphi-B CMCC 50094), *Listeria monocytogenes* (*L. monocytogenes* ATCC 19111), *Bacillus cereus* (*B. cereus* CMCC 63303) and two *B. cereus* clinical isolates (*B. cereus*-1, *B. cereus*-2, obtained from Xi’an Center for Disease Control and Prevention). All strains were cultured in Luria-Bertani (LB) broth (Beijing Land Bridge, China) at 37 °C with continuous shaking at 200 rpm for 12–16 h. Bacterial genomic DNA was extracted using the Bacteria Genomic DNA Kit (ZOMANBIO, Beijing, China) and stored at −20 °C until further use.

### 2.2 Primer design

To design specific primers for *Salmonella* spp., the *invA* gene sequences from multiple *Salmonella* strains were aligned to identify conserved regions. Three pairs of primers targeting the conserved region of the *invA* gene were designed using Primer Premier 5 software (Supplementary Table S1). Both PCR and RPA were employed to screen for primers exhibiting optimal sensitivity and specificity. To enhance G4 structure formation in the amplified products, tandem cytosine repeats (5′-CCCACCCACCCACCC-3′) were appended to the 5′ end of the primers based on previous studies showing that their complementary sequences can form stable G4 structures. All primers were synthesized by Sangon Biotech (Shanghai, China) and are listed in Supplementary Table S1.

### 2.3 RPA reaction

RPA reactions were performed using the TwistAmp Basic Kit (TwistDX, Cambridge, United Kingdom) in a total reaction volume of 50 μL, strictly following the manufacturer’s instructions. The reaction mixture consisted of 29.5 μL of Primer-Free Rehydration buffer, 382 ng of bacterial genomic DNA, 0.48 μM of each primer, and 12.2 μL of nuclease-free water (ddH_2_O). The mixture was thoroughly vortexed and briefly centrifuged, then transferred into freeze-dried reaction tubes, followed by the addition of 2.5 μL of 280 mM magnesium acetate (MgOAc) to initiate the reaction. The reaction tubes were immediately incubated at 39 °C in a constant-temperature heating block for 30 min and then heated at 95 °C for 3 min to terminate the reaction.

### 2.4 G4 peroxidase assay

The formation of stable G4 structures from duplex DNA requires potassium ions (K^+^) (Li et al., 2010). A potassium-rich buffer was prepared containing 20 mM Tris–HCl (pH 7.4), 200 mM KCl, and 0.1% (v/v) TritonX-100, and sterilized by filtration through a 0.22 μm membrane. For DNAzyme assembly, 5 μL of the RPA amplification product, 5 μL of 5 μM hemin solution (dissolved in DMSO), and 10 μL of K^+^-rich buffer were mixed, then heated at 95 °C for 3 min and immediately cooled on ice for 5 min to facilitate G4/hemin DNAzyme formation as described previously (Kong et al., 2010; Jia et al., 2012; Li et al., 2024). Negative controls were established using RPA reaction mixtures without DNA templates, and all experiments were performed in triplicate.

The peroxidase-like activity of the G4/hemin DNAzyme was assessed via a chromogenic assay using ABTS as the substrate as previously described (Kong et al., 2010; Jia et al., 2012; Li et al., 2024). Briefly, 5 μL of substrate solution (containing ABTS, ATP and H_2_O_2_) was added to 20 μL of the assembled G4/hemin DNAzyme mixture, yielding final concentrations of 10 mM ABTS, 8 mM ATP and 36 mM H_2_O_2_. After incubation for 10–20 min at room temperature, peroxidase activity was evaluated by direct visual observation of color development and quantified by measuring absorbance at 420 nm using a microplate reader.

### 2.5 Optimization of reaction conditions

To maximize the efficiency of RPA amplification for sensitive chromogenic detection, key reaction parameters were systematically optimized. For RPA optimization, three variables were evaluated: reaction temperatures (37 °C, 39 °C, 42 °C), primer concentrations (0.24 μM, 0.48 μM, 0.96 μM), and incubation time (20 min, 30 min, 40 min). For the chromogenic system, the concentrations of four critical components were tested: hemin (0.01 μM, 0.1 μM, 1 μM, and 10 μM), ABTS (10 mM, 20 mM, 40 mM, and 80 mM), H_2_O_2_ (9 mM, 36 mM, 72 mM, 108 mM, and 144 mM) and ATP (0, 2 mM, 4 mM, 8 mM, and 10 mM). Deionized water served as the template for negative control reactions throughout the optimization process, and each condition was tested in triplicate. Optimization results were assessed based on the intensity of the green color (visual observation) and the absence of false-positive signals in negative controls.

### 2.6 Specificity test

The specificity of the assay was evaluated using genomic DNA extracted from *Salmonella* spp. (target) and four common non-target foodborne pathogens: *E. coli*, *S. flexneri*, *L. monocytogenes* and *E. cloacae*. All DNA templates were adjusted to a uniform concentration of 100 ng/μL before use. The RPA-G4 chromogenic assay was conducted under optimized conditions, and the specificity was determined by the presence or absence of a green color. Each test was independently repeated three times.

### 2.7 Validation with artificially contaminated food samples and sensitivity test

The applicability and sensitivity of the developed RPA-based detection system were evaluated using artificially contaminated milk samples (confirmed *Salmonella*-free by the national standard method GB 4789.4-2016) as described previously (Zhao et al., 2021). A single colony of *Salmonella* spp. was inoculated into the milk, followed by serial 10-fold dilutions to establish a contamination gradient. The initial bacterial concentration was calculated by colony-forming unit (CFU) counting to be 1.2 × 10^9^ CFU/mL. For artificial contamination, 1 mL of each diluted bacterial suspension was added to 9 mL of sterilized milk, yielding contamination levels ranging from 1.2 × 10^5^ to 1.2 CFU/mL. The contaminated milk samples underwent thermal lysis at 95 °C for 8 min, and 1 μL of the sample was directly used as the template in the RPA reaction, followed by chromogenic qualitative detection. Sterile milk without bacterial contamination served as the negative control. The limit of detection was defined as the lowest bacterial concentration that produced an absorbance value significantly higher than the blank control (0 CFU/mL) (Mean + 3SD, *p* < 0.05).

### 2.8 Statistical analysis

All experiments were performed in triplicate. Statistical analysis was conducted using SPSS 26.0 software. Student’s *t*-test was used to compare two groups, and *p* < 0.05 was considered statistically significant.

## 3 Results

### 3.1 Principle of the designed RPA-G4 assay

**Figure 1:**
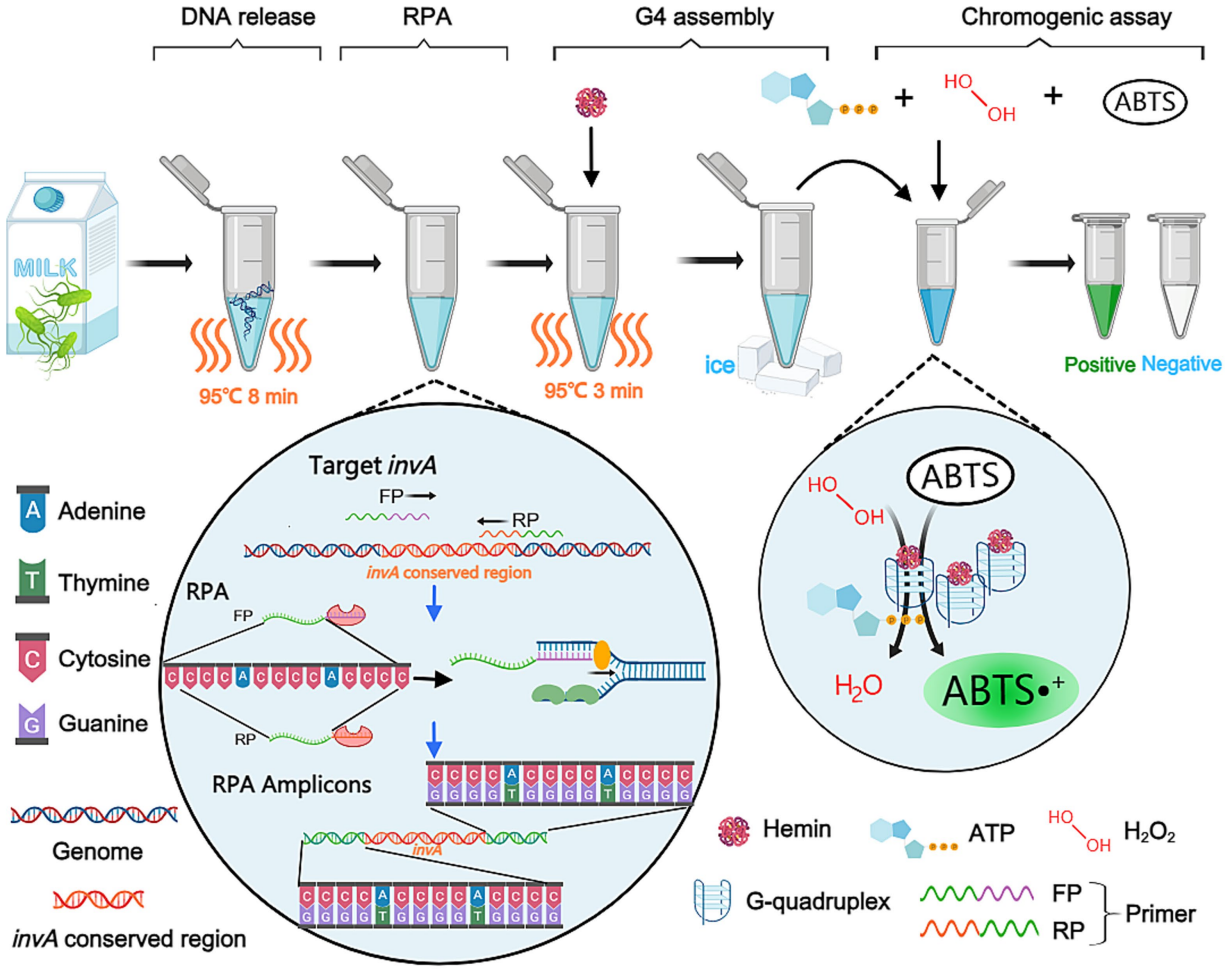
Scheme for rapid detection of *Salmonella* spp. based on RPA and G4/hemin DNAzyme catalysis (RPA-G4).

The detailed mechanism of the RPA-G4 assay is illustrated in Figure 1. A primer set targeting the conserved *invA* gene of *Salmonella* spp. enables amplification across diverse *Salmonella* serotypes. In the presence of *Salmonella* contamination, genomic DNA templates are first released by thermal lysis (95 °C for 8 min) and subsequently amplified by RPA using the engineered primers, yielding dsDNA amplicons. A shown in the RPA amplification schematic of Figure 1, the engineered primers (FP and RP) contain tandem cytosine repeats (5′-CCCACCCACCCACCC-3′) at their 5′ ends. During RPA amplification, the complementary strand of the cytosine-rich segment is synthesized, resulting in all amplicons harboring guanine-rich sequences (5′-GGGTGGGTGGGTGGG-3′) at their 3′ ends. Previous studies have shown that this sequence can fold into a stable G4 structure and bind hemin to form a G4/hemin DNAzyme complex. As depicted in the chromogenic assay schematic, this complex exhibits peroxidase-like catalytic activity, catalyzing the H_2_O_2_-mediated oxidation of 2,2′-azino-bis(3-ethylbenzothiazoline-6-sulfonic acid) diammonium salt (ABTS). Following G4 assembly under ice incubation, this catalytic reaction produces a visible color change (from colorless to green) that directly indicates the presence of *Salmonella* spp. Beyond target amplification by RPA, each G4/hemin DNAzyme complex further amplifies the signal by continuously catalyzing the oxidation of multiple ABTS molecules through catalytic turnover, thereby further enhancing detection sensitivity. In summary, the proposed RPA-G4 system achieves dual signal amplification—target amplification via RPA and catalytic signal enhancement via G4/hemin DNAzyme—greatly improving detection sensitivity compared with traditional amplicon detection methods and enabling instrument-free, naked-eye detection of *Salmonella* spp.

### 3.2 Primer screening and validation

**Figure 2:**
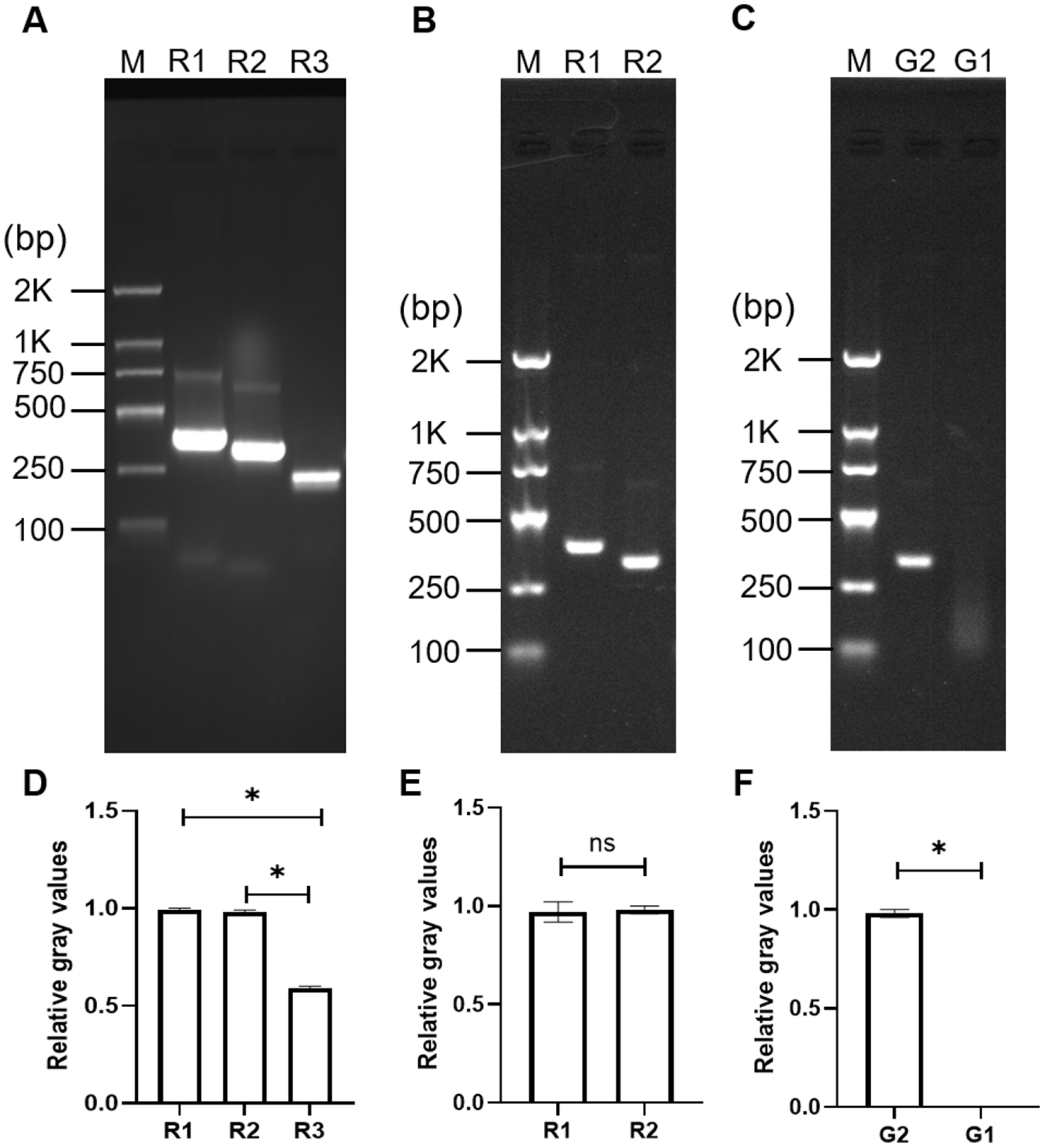
Identification of optimal specific primers for detection of *Salmonella* spp. Agarose gel electrophoresis of conventional PCR amplicons **(A)**, RPA amplicons **(B,C)** using candidate primers; quantification of the agarose gel electrophoresis images using Image J software **(D–F)**. Error bars represent standard deviation (SD, *n* = 3). *p < 0.05, ns: non significance; M: DNA Marker.

The *invA* gene, a conserved marker for *Salmonella* spp. was selected as the target due to its high diagnostic value (Kasturi and Drgon, 2017; Ye et al., 2019). To further confirm its conservation and ensure the detection of diverse *Salmonella* serotypes, BLAST analysis of the *invA* gene on the NCBI database was performed. The results revealed that the *invA* gene was highly conserved among different *Salmonella* species, including *S*. Typhimurium, *S.* Dublin and *S*. Enteritidis, with more than 95% sequence identity across all tested serotypes. Based on these findings, three pairs of primers (R1, R2, R3; listed in Supplementary Table S1) targeting the conserved region of the *invA* gene were designed using Primer Premier 5 software to minimize primer-dimer formation. Initial screening via conventional PCR identified R1 and R2 as optimal candidates, based on their superior amplification efficacy indicated by higher band intensities compared to R3 (Figures 2A,D). Band intensities were quantified using Image J software (*n* = 3, mean ± SD). Statistical analysis showed that the relative gray values of R1 and R2 were 0.99 ± 0.01 and 0.98 ± 0.01, respectively, which were significantly higher than that of R3 (0.59 ± 0.01, *p* < 0.05), confirming their superior amplification efficacy. Subsequent RPA validation confirmed that both R1 and R2 successfully amplified the target region, generating bands of the expected size (Figures 2B,E). The RPA-amplified products of R1 and R2 were extracted, purified and verified by Sanger sequencing. The sequencing results were highly consistent with the target *invA* gene sequence, confirming that the amplified fragments were indeed the intended Salmonella-specific sequence (data not shown). To enable G4 structure formation, tandem cytosine repeats were added to the 5′-termini of these primers, yielding modified primer sets G1 and G2. However, only G2 produced a detectable RPA product (Figures 2C,F), possibly because the addition of tandem cytosine repeats to R1 may have affected primer binding efficiency. Consequently, G2 was selected as the optimal primer set for the downstream experiments.

### 3.3 RPA conditions optimization

To maximize the efficiency of G4 structure formation and downstream chromogenic detection, RPA reaction conditions were systematically optimized using the G2 primers. Reagents were sourced from the TwistAmp Basic Kit, following the manufacturer’s protocol, with optimization focused on three key parameters: primer concentration, reaction temperature, and incubation time.

**Figure 3:**
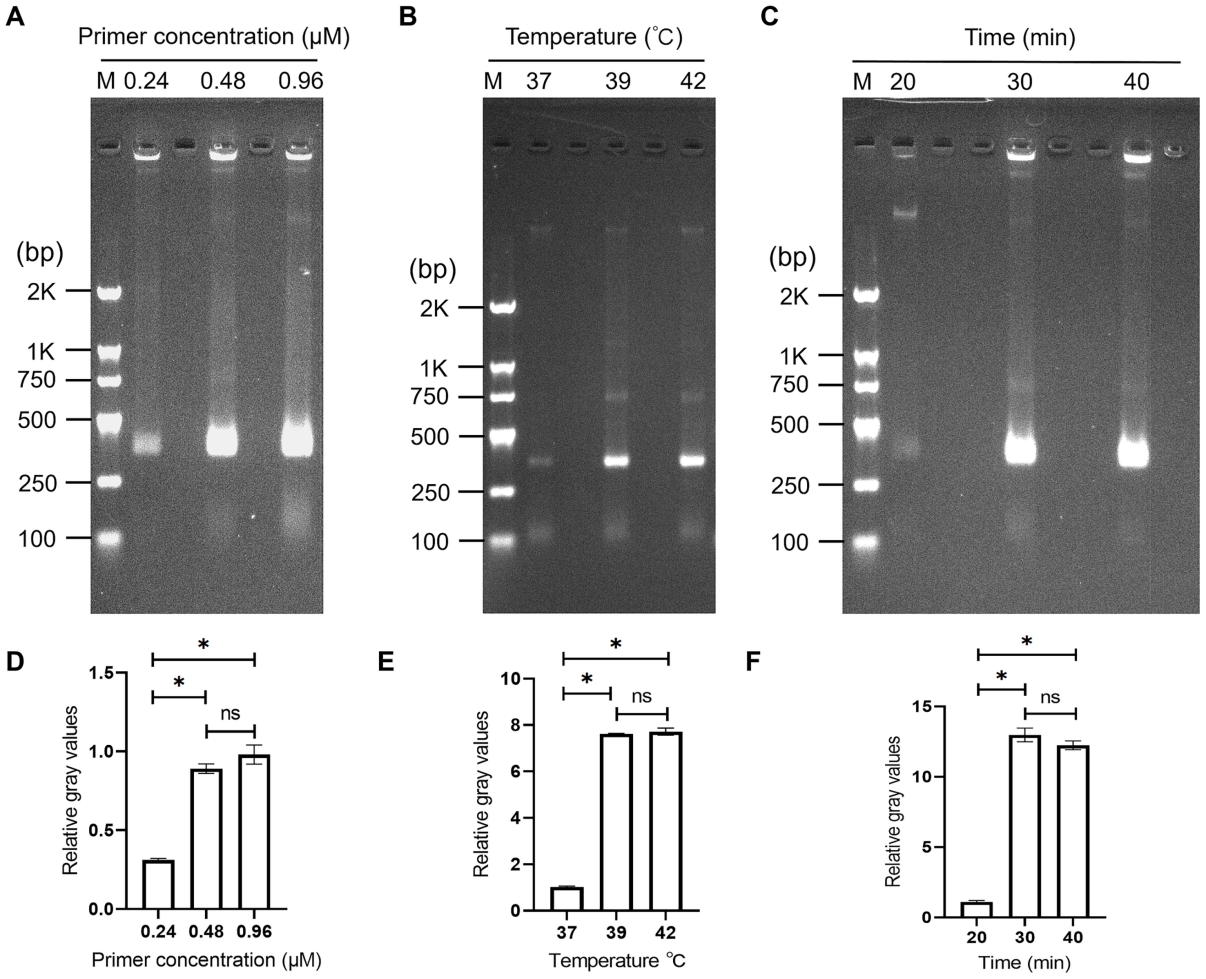
Optimization of RPA conditions, including primer concentration **(A,D)**, reaction temperature **(B,E)** and time **(C,F)**. **(A–C)** Agarose gel electrophoresis resuts of RPA amplicons; corresponding band quantification results of **(A–C)** using Image J software. All values were expressed as mean ± SD (standard deviation, *n* = 3), *p < 0.05. M: DNA Marker.

First, primer concentration was optimized to balance detection performance and experimental cost. Concentrations of 0.24 μM, 0.48 μM, and 0.96 μM were tested, with a fixed incubation time of 30 min and temperature of 39 °C. Band intensities of Figure 3A were quantified using ImageJ software (National Institutes of Health, United States) (*n* = 3, mean ± SD). As shown in Figures 3A,D, the lowest amplification efficacy was observed at 0.24 μM (relative gray value: 0.31 ± 0.01), whereas comparable and significantly higher amplification efficiencies were achieved at 0.48 μM (gray value 0.89 ± 0.03) and 0.96 μM (gray value 0.98 ± 0.06). There was no significant difference between 0.48 μM and 0.96 μM primer concentrations (*p* > 0.05). Accordingly, 0.48 μM was selected as the optimal working concentration, balancing detection performance and experimental cost while avoiding unnecessary primer waste.

Subsequently, reaction temperature was optimized using the determined optimal primer concentration (0.48 μM) and a fixed incubation time of 30 min. Reaction temperatures of 37 °C, 39 °C and 42 °C were evaluated. As shown in Figures 3B,E, quantitative analysis revealed significantly lower amplification efficiency at 37 °C (1.03 ± 0.03) compared to 39 °C (7.62 ± 0.20, *p* < 0.05) and 42 °C (7.72 ± 0.15, *p* < 0.05). No significant difference in amplification efficiency was observed between 39 °C and 42 °C (*p* > 0.05). Considering the practical applicability for on-site detection—where 39 °C is more readily achievable without high-precision temperature-control than 42 °C—39 °C was selected as the optimal reaction temperature.

Finally, incubation time was optimized using the optimal temperature (39 °C) and primer concentration (0.48 μM) to balance reaction efficiency and detection speed. Results showed that the band intensity increased with time, reaching a plateau at 30 min (gray value: 12.98 ± 0.49), with no significant increase at 40 min (12.24 ± 0.31, *p* > 0.05) (Figures 3C,F). Thus, 30 min was selected as the optimal reaction time to shorten the detection cycle without compromising amplification efficiency.

### 3.4 Chromogenic reaction conditions optimization

To achieve optimal detection performance and fully exploit the catalytic signal amplification of the G4/hemin DNAzyme system, critical parameters—including hemin, ABTS, H_2_O_2_ and ATP concentrations—were carefully optimized. All experiments were performed in triplicate. Color development was quantified by measuring absorbance at 420 nm, providing objective data for condition optimization and accurately reflecting the efficiency of G4/hemin-mediated signal amplification.

**Figure 4:**
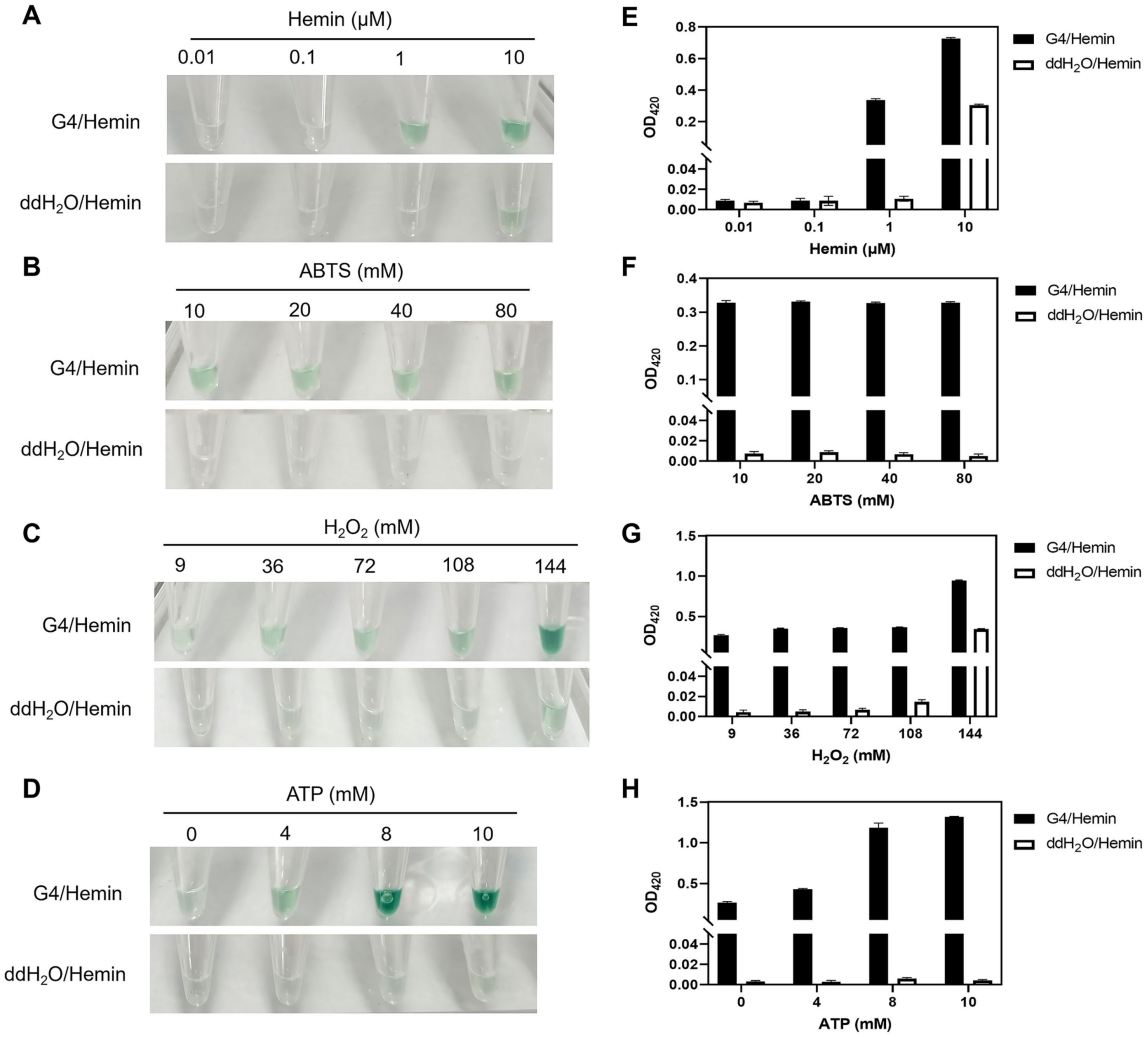
Effects of hemin **(A,E)**, ABTS **(B,F)**, H_2_O_2_ **(C,G)**, and ATP **(D,H)** concentrations on the analytical performance of G4/hemin DNAzyme. **(A–D)** Visual chromogenic images of the catalytic reaction; **(E–H)** corresponding absorbance values measured at 420 nm using a microplate reader. All the experiments were replicated three times. Error bars represent standard deviations (SD, *n* = 3).

Hemin concentrations ranging from 0.01 μM to 10 μM were tested to maximize color differentiation between positive and negative samples and optimize G4/hemin DNAzyme signal amplification. As shown in Figures 4A,E, color development in positive samples intensified with increasing hemin concentration, accompanied by a gradual rise in absorbance at 420 nm, indicating enhanced catalytic activity and more efficient signal amplification. However, obvious background coloration and elevated baseline absorbance were observed in the negative control at 10 μM. Therefore, 1 μM hemin was selected as the optimal concentration to balance signal intensity, low background and efficient catalysis.

For chromogenic substrates, each parameter was optimized independently while keeping other conditions constant. ABTS concentration (10–80 mM) exerted only minor effects on absorbance at 420 nm and visual color development (Figures 4B,F). H_2_O_2_ concentrations above 144 mM induced false-positive signals and high background absorbance, whereas 9 mM H_2_O_2_ yielded negligible color response and absorbance (Figures 4C,G). Thus, 10 mM ABTS and 36 mM H_2_O_2_ were selected as optimal concentrations for the chromogenic reaction.

Previous studies have reported that ATP stabilizes the ABTS·^+^ chromogenic signal by inhibiting its disproportionation (Kong et al., 2010; Jia et al., 2012). In this study, increasing ATP concentrations enhanced the color intensity of positive samples and absorbance at 420 nm, with no significant difference observed between 8 mM and 10 mM (Figures 4D,H). Accordingly, 8 mM ATP was chosen to ensure long-term signal stability and maintain the persistent signal amplification effect of the G4/hemin DNAzyme system.

### 3.5 Sensitivity and specificity examination

**Figure 5:**
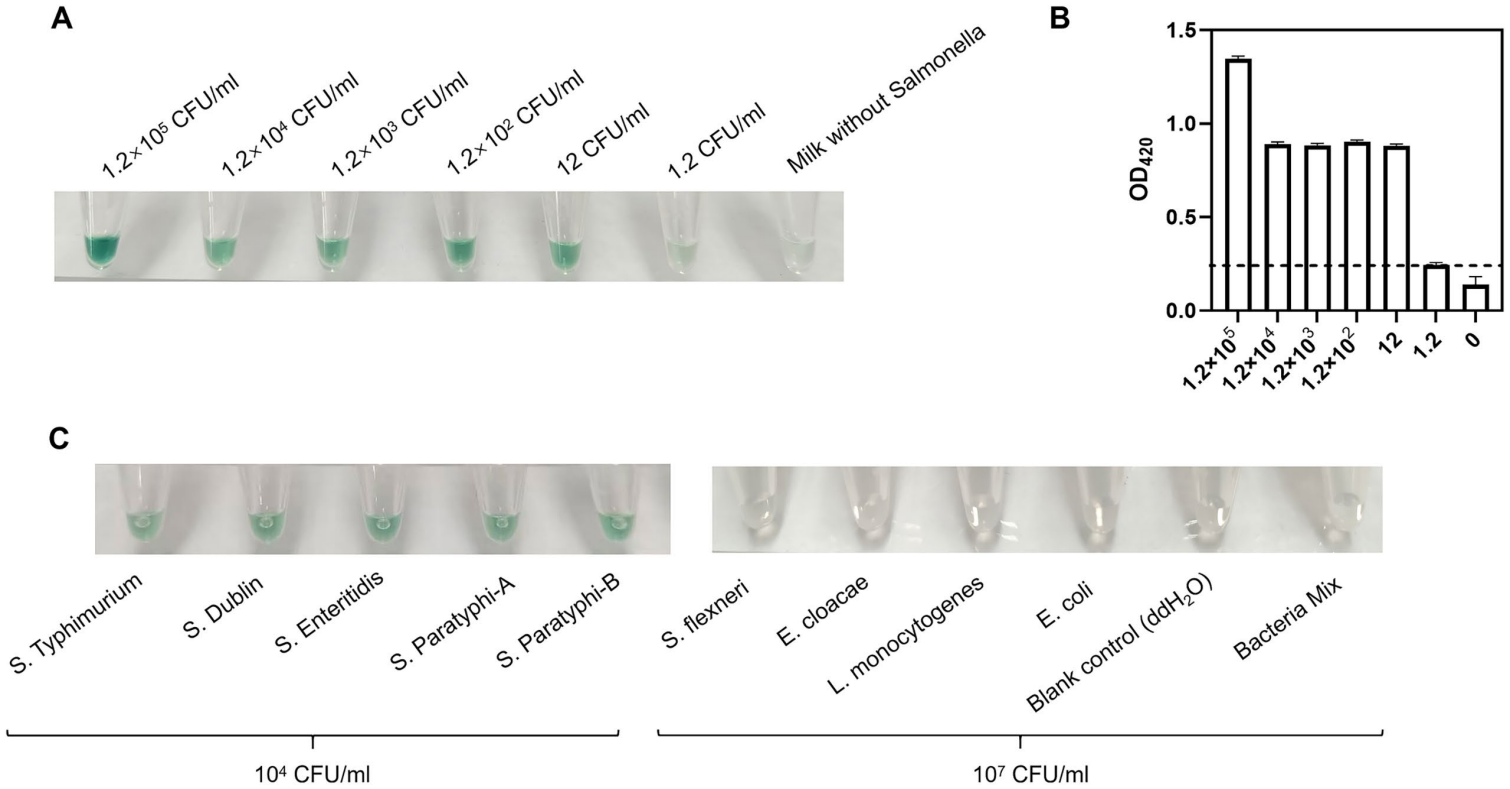
Sensitivity **(A,B)** and specificity **(C)** test of RPA-G4 assay. **(A)** Visual chromogenic images for detecting *Salmonella* spp. in range from 1.2 × 10^5^ to 0 CFU/mL. **(B)** Absorbance values at 420 nm analyzed using a microplate reader. **(C)** Specificity test of the RPA-G4 assay with *S*. Typhimurium, *S.* Dublin, *S*. Enteritidis, *S.* Paratyphi-A, *S*. Paratyphi-B, *S. flexneri*, *E. cloacae*, *L. monocytogenes*, and *E. coli*. All the experiments were replicated three times. Error bars represent standard deviations (SD, *n* = 3).

To evaluate the sensitivity of the method, artificially contaminated milk containing *Salmonella* spp., prepared via serial 10-fold dilutions, was tested under optimal conditions. Notably, complex genomic DNA extraction was unnecessary; simple thermal lysis (95 °C for 8 min) was sufficient for downstream RPA and chromogenic reaction. As shown in Figure 5A, the green color gradually diminished as *Salmonella* concentration decreased from 1.2 × 10^5^ CFU/mL to 0 CFU/mL. Color intensity was quantified by absorbance at 420 nm (Figure 5B). The detection threshold was defined as the value of the blank control (0 CFU/mL) plus three standard deviations (indicated by the dotted line in Figure 5B). Results showed that 12 CFU/mL *Salmonella* spp. could induce a distinguishable green color change from the blank control, which was confirmed by statistical analysis, indicating a limit of detection (LOD) of 12 CFU/mL for this method (Figure 5B). In Table 1, a series of reported molecular methods for *Salmonella* detection have been exhibited. Regarding sensitivity, this method exhibits higher sensitivity than most existing methods including qPCR (Ding et al., 2017), digital PCR (Wang et al., 2018) and all listed RPA-based assays. The high sensitivity can be attributed to the introduction of the RPA amplification into the G4/hemin DNAzyme catalysis, which efficiently translates target amplification into visible color signals and further enhances the output signal.

**Table 1:** Comparison of the performance of molecular methods for *Salmonella* spp. detection.

Methods	LOD (CFU/ml)	Labels	Time (h)	Themo cycler	References
PCR-dot hybridization	10^3^	No	7	Yes	Li et al. (2000)
qPCR	10^2^	No	2	Yes	Ding et al. (2017)
Multiplex PCR	10^2^	No	4	Yes	Paião et al. (2013)
Digital PCR	10^2^	Yes	<2	Yes	Wang et al. (2018)
RPA-LFD	140	Yes	<1	No	Zhao et al. (2021)
LAMP	200	Yes	1	No	Kaur et al. (2018)
RPA-CRISPR/Cas12a	50	Yes	1	No	Mao et al. (2022)
RPA-PCA	5 × 10^3^	No	1	No	Li X. et al. (2021)
RPA-CRISPR/Cas9-LFD	100	Yes	1	No	Wang et al. (2022)

RPA, recombinase polymerase amplification; LFD, lateral flow dipstick; LAMP, loop-mediated isothermal amplification; PCA, photosensitization colorimetric assay.

To investigate the specificity of the established method, five *Salmonella* strains at 10^4^ CFU/mL and four common foodborne pathogens at 10^7^ CFU/mL (*E. coli*, *S. flexneri*, *L. monocytogenes* and *E. cloacae*) were tested. As shown in Figure 5C, a green color appeared only in the presence of *S*. Typhimurium, *S.* Dublin, *S. enterica*, *S*. Paratyphi-A and *S*. Paratyphi-B, while non-*Salmonella* strains produced no observable color change. These results confirm the high specificity of the method, demonstrating no cross-reactivity with non-target bacterial species.

### 3.6 Application to *Bacillus cereus* detection

**Figure 6:**
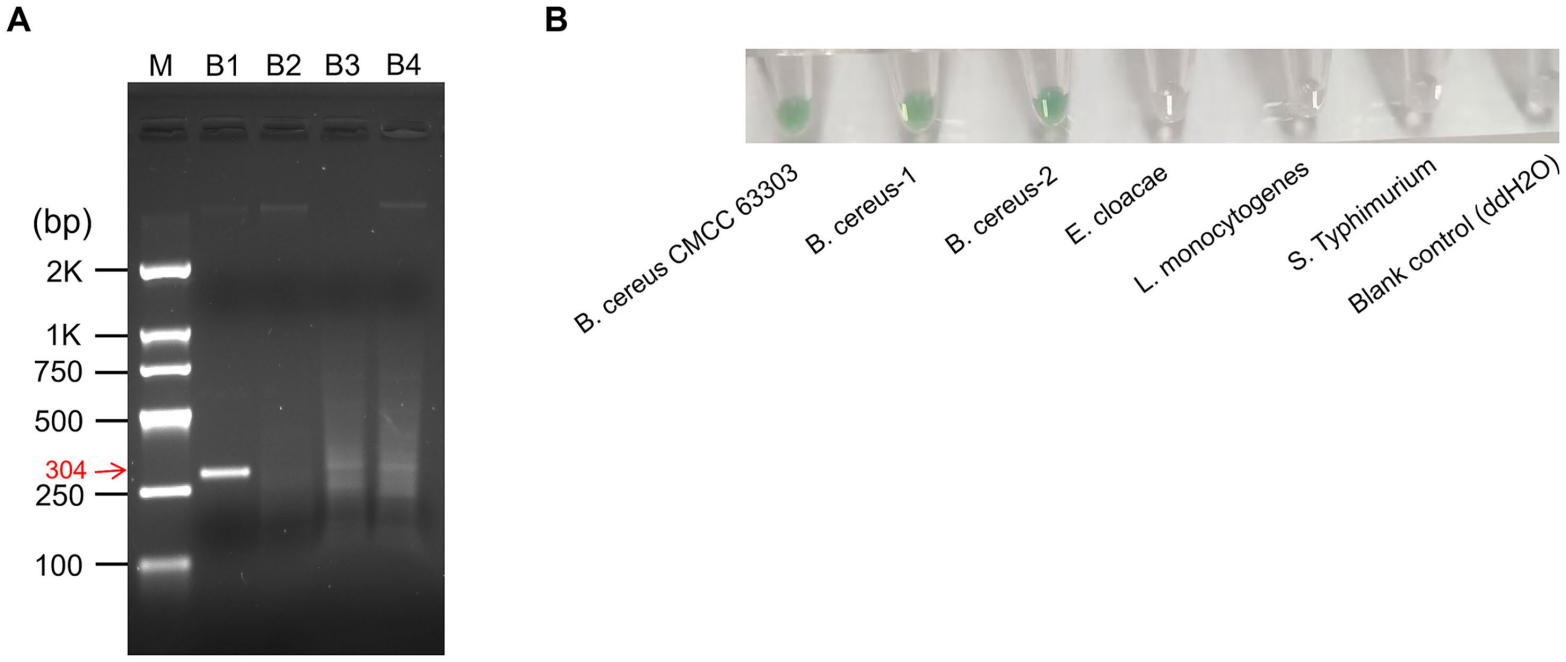
Application of the RPA-G4 assay for *B. cereus* detection. **(A)** Identification of optimal primers for *B. cereus* detection via agarose gel electrophoresis. **(B)** Specificity test of RPA-G4 assay for *B. cereus* detection. All experiments were performed in triplicate.

The rapid visual detection method proposed in this study can be further extended to *B. cereus* detection, another prevalent foodborne pathogen known for toxin production. Despite its pathogenic potential, the global epidemiology of *B. cereus* remains underreported, partly due to the lack of rapid diagnostic methods in clinical and field settings (Dietrich et al., 2021; Enosi Tuipulotu et al., 2021). Four pairs of primers (designated B1, B2, B3 and B4, Supplementary Table S1) incorporating tandem cytosine repeats (consistent with the design principle of *Salmonella* primers) targeting the *nheA* gene of *B. cereus* were designed and evaluated for their amplification efficacy (Figure 6A). The *nheA* gene encodes the non-hemolytic enterotoxin, which is a conserved marker for *B. cereus*. Only B1 yielded an obvious RPA amplicon with clear band; no visible amplification products were observed for B2, B3 or B4. Thus, primer B1 was selected as the optimal *B. cereus*-specific primer for subsequent detection.

To further validate the applicability and specificity of the established detection method for *B. cereus*, a standard *B. cereus* strain and two food-isolated strains (belonging to ST-26 and ST-24 genotypes, isolated from rice samples) were tested. Meanwhile, three non-*B. cereus* strains—*E. cloacae*, *L. monocytogenes* and *Salmonella* spp.—were included as controls to assess cross-reactivity. As shown in Figure 6B, obvious green coloration was observed for all three *B. cereus* strains, confirming the successful detection of *B. cereus*. In contrast, no positive signal was detected for *E. cloacae*, *L. monocytogenes* and *Salmonella* spp. These findings demonstrate that the proposed RPA-G4 detection method is applicable not only to Gram-negative *Salmonella* spp. but also to Gram-positive *B. cereus*, including both standard and food-isolated strains of diverse genotypes. Moreover, the method exhibits no cross-reactivity with other common foodborne pathogens, ensuring reliability in practical applications. Collectively, these results confirm that by simply modifying the target-specific primers, the method can be extended to the detection of various foodborne pathogens, providing a universal strategy for on-site food safety surveillance.

## 4 Discussion

Foodborne diseases caused by *Salmonella* spp. remain a widespread and persistent public health concern globally. Ingestion of contaminated food or water can lead to clinical symptoms ranging from acute gastroenteritis to severe systemic infections, including septicemia and typhoid fever (Fleckenstein et al., 2021). Timely and reliable detection of *Salmonella* spp. is therefore critical for early warning, epidemiological surveillance, and effective control of foodborne outbreaks.

Traditional culture-based detection methods, although highly specific, are time-consuming and labor-intensive, usually requiring 3–7 days for definitive results. PCR-based technologies, including PCR, qPCR and digital PCR, have substantially improved detection speed and sensitivity. However, their reliance on expensive thermal cyclers and professional personnel restricts their use in resource-limited regions and field settings (Faraji et al., 2018; Lei et al., 2021). Against this backdrop, isothermal amplification techniques such as RPA have gained increasing attention for point-of-care testing (POCT), owing to their rapid amplification at constant temperature (37–42 °C) without complex thermal cycling equipment.

Despite the rapid development of RPA-based detection systems, several bottlenecks continue to limit their practical application. Conventional RPA usually relies on post-amplification analysis such as agarose gel electrophoresis, which are time-consuming and require additional instruments. RPA combined with LFD enables visual detection, but usually necessitates the labeling of primers and probes (e.g., with biotin or fluorescent tags), which increases cost and complicates probe design (Du et al., 2018; Gao et al., 2018; Li et al., 2019; Wu et al., 2020; Mao et al., 2022; Wang et al., 2022; Wang et al., 2023). To address these limitations, this study established a novel RPA-G4 chromogenic assay based on G4/hemin DNAzyme catalysis for the rapid, sensitive and specific detection of *Salmonella* spp.

A key innovation of the proposed RPA-G4 system is its label-free and modification-free design, which greatly simplifies detection and reduces cost. Unlike RPA-LFD or fluorescent RPA assays that require chemically modified primers or probes (Du et al., 2018; Zhao et al., 2021; Li et al., 2022; Wang et al., 2022), the RPA-G4 system incorporates only a short cytosine-rich tandem repeat sequence without biotin, antigen, or fluorescent labeling. This design simplifies primer synthesis, lowers cost, and enhances stability and reproducibility. Moreover, the results are directly observable by the naked eye through a distinct green color change, eliminating the need for analytical instruments, rendering the method highly suitable for POCT and field detection in low-resource areas.

Another key innovation of the RPA-G4 system lies in the dual signal amplification mechanism, which underpins the assay’s exceptional sensitivity. The first layer of the amplification is achieved through efficient isothermal amplification via RPA, which rapidly generates abundant target amplicons. The second layer stems from the catalytic turnover activity of G4/hemin DNAzyme, wherein each G4/hemin structure continuously catalyzes the oxidation of multiple ABTS molecules, generating a strongly amplified chromogenic signal. This dual-amplification design has been demonstrated to significantly improve detection sensitivity in previous reports (Li et al., 2024; Yuan et al., 2025), and our findings are in agreement with these observations.

In addition, RPA has been widely reported to exhibit stronger tolerance to background DNA and various PCR inhibitors such as humic acids, polysaccharides, and food matrix components compared to other isothermal amplification methods like loop-mediated isothermal amplification (LAMP) and nucleic acid sequence-based amplification (NASBA) (Daher et al., 2016; Li et al., 2019; Liu et al., 2019). This robust anti-interference capacity enables efficient amplification even in complex food matrices without rigorous DNA purification, which further contributes to the high sensitivity and reliability of the assay in real-world samples.

This dual-amplification strategy, combined with the inherent anti-inhibitor capacity of RPA, enables the assay to achieve an outstanding LOD of 12 CFU/mL in artificially contaminated milk samples surpassing the sensitivity of most conventional PCR and previously reported RPA-based methods. Our LOD is comparable or superior to those of recently published visual detection systems for *Salmonella* (e.g., RPA-LFD, CRISPR-based assays), supporting the high sensitivity of the current platform (Li J. et al., 2021; Mao et al., 2022; Wang et al., 2022). Such high sensitivity is particularly valuable for detecting low-level pathogenic contamination in food, supporting early warning and prevention of foodborne diseases.

Furthermore, the RPA-G4 platform demonstrates excellent versatility and expandability. By replacing *Salmonella*-specific *invA* primers with primers targeting the *nheA* gene of *B. cereus*, the system was successfully adapted for the visual detection of *B. cereus* without altering the core reaction mechanism or chromogenic readout. This result confirms that the RPA-G4 strategy is not restricted to a single pathogen but can serve as a universal visual detection platform for diverse pathogens. This flexible molecular design provides a convenient and generalizable approach for developing rapid detection assays for other pathogenic microorganisms, highlighting its broad application potential in food safety monitoring.

Nevertheless, several limitations of the current system warrant acknowledgment. The RPA-G4 assay is designed for qualitative analysis, clearly indicating the presence or absence of target pathogens but not supporting accurate quantification of pathogen load. For food safety assessment and clinical diagnosis, quantitative detection is essential to evaluate contamination levels and potential infection severity. Therefore, future research will optimize the reaction system to establish a linear relationship between 420 nm and bacterial concentration, enabling semi-quantitative detection.

The RPA-G4 relies on the catalytic activity of G4/hemin DNAzyme. With good thermal stability, low cost, and simple operation, G4/hemin holds promising application prospects in bioanalytical and biomedical fields (Li et al., 2011; Atsumi and Belcher, 2018; Peng et al., 2018). However, the catalytic activity of traditional G4/hemin DNAzyme is highly susceptible to interference from complex environmental conditions. Although the RPA-G4 assay was successfully validated in artificially contaminated milk samples in this study, *Salmonella* is not only widely distributed in milk but also requires routine detection in samples with more complex matrices, such as meat and eggs. In our preliminary experiments, we attempted to directly apply this method to the detection of meat and egg samples. The results demonstrated that RPA could amplify the *invA* gene to produce obvious bands, whereas no significant green color change was observed in the chromogenic reaction (data not shown). This phenomenon indicates that the detection performance of the traditional G4/hemin DNAzyme is insufficient and needs further improvement to adapt to complex sample matrices. In future research, we will attempt to construct a zipper-G4/hemin system with reference to previous relevant studies (Li J. et al., 2021). The zipper-G4/hemin system is constructed through hybridization of the complementary G4 sequences and DNA-grafted hemin probes, which mimics the tight spatial configuration of the cofactor and apoenzyme in natural proteases, endowing the DNAzyme with higher catalytic efficiency, faster catalytic rate, and stronger environmental tolerance than the classical G4/hemin DNAzyme. Therefore, it can effectively resist interference from complex food matrices and improve detection performance in actual samples.

In conclusion, this study successfully established a label-free, instrument-free, rapid, sensitive and specific chromogenic RPA assay based on G4/hemin DNAzyme catalysis for the detection of *Salmonella* spp. The dual signal amplification mechanism confers high sensitivity, while the label-free primer design and visual readout ensure low cost and ease of use. The entire detection process can be completed within 1 h. Furthermore, the platform can be readily extended to other foodborne pathogens, demonstrating strong universality. This method provides a promising and practical tool for the rapid detection of *Salmonella* and other pathogens, especially in resource-limited settings.

## Data Availability

The original contributions presented in the study are included in the article/Supplementary material, further inquiries can be directed to the corresponding author.
