# Supplementary material for: Development of a recombinase polymerase amplification-based chromogenic assay for rapid detection of Salmonella
Source: Front Microbiol. 2026 Apr 10;17:1819643. doi: 10.3389/fmicb.2026.1819643 (PMC13106402; doi:10.3389/fmicb.2026.1819643)
Supplement: Supplementary file 1 [file Table_1.docx]

***Supplementary materials***

**Table S1 Sequences of *Salmonella* and *B. cereus* primers**

| **Primer Name** | **Genes** | **Sequence (5’-3’)** | **Fragment length** |
| --- | --- | --- | --- |
| R1 | *invA* | Forward primer: AAAAGAAGGGTCGTCGTTAGGACTGATTGG | 382 bp |
|  |  | Reverse primer:  GAGTTTCTCCCCCTCTTCATGCGTTACCCA |  |
| R2 |  | Forward primer:  GACCGTACCGTTGATATTACTTGTGCCGAA | 328 bp |
|  |  | Reverse primer:  GAGTTTCTCCCCCTCTTCATGCGTTACCCA |  |
| R3 |  | Forward primer:  AGTATTTCTGGGTAACGCATGAAGAGGGGGA | 217 bp |
|  |  | Reverse primer:  TGTACCGTGGCATGTCTGAGCACTTCTTTAA |  |
| G1 |  | Forward primer:  CCCACCCACCCACCC  AAAAGAAGGGTCGTCGTTAGGACTGATTGG | 412 bp |
|  |  | Reverse primer:  CCCACCCACCCACCC  GAGTTTCTCCCCCTCTTCATGCGTTACCCA |  |
| G2 |  | Forward primer:  CCCACCCACCCACCC  GACCGTACCGTTGATATTACTTGTGCCGAA | 358 bp |
|  |  | Reverse primer:  CCCACCCACCCACCC  GAGTTTCTCCCCCTCTTCATGCGTTACCCA |  |
| B1 | *nheA* | Forward primer:  CCCACCCACCCACCC  GCAAACAGAAGTGAAAACAGTATATGCGCAA | 304 bp |
|  |  | Reverse primer:  CCCACCCACCCACCC  TCCTGCTAGTTCATAGAGCTTACTATAATAG |  |
| B2 |  | Forward primer:  CCCACCCACCCACCC  CCCACCCACCCACCCTACGCTAAGGAGGGGCAA | 246 bp |
|  |  | Reverse primer:  CCCACCCACCCACCC  CCCACCCACCCACCCCCACTCTCGCACATTCGC |  |
| B3 |  | Forward primer:  CCCACCCACCCACCC  CCCACCCACCCACCCACAGGGTTATTGGTTAC | 491 bp |
|  |  | Reverse primer:  CCCACCCACCCACCC  CCCACCCACCCACCCATACTCTCTTGGATGCT |  |
| B4 |  | Forward primer:  CCCACCCACCCACCC  CCCACCCACCCACCCTACGCTAAGGAGGGGCAAA | 251 bp |
|  |  | Reverse primer:  CCCACCCACCCACCC  CCCACCCACCCACCCTCAATCCACTCTCGCACAT |  |

Fig S1


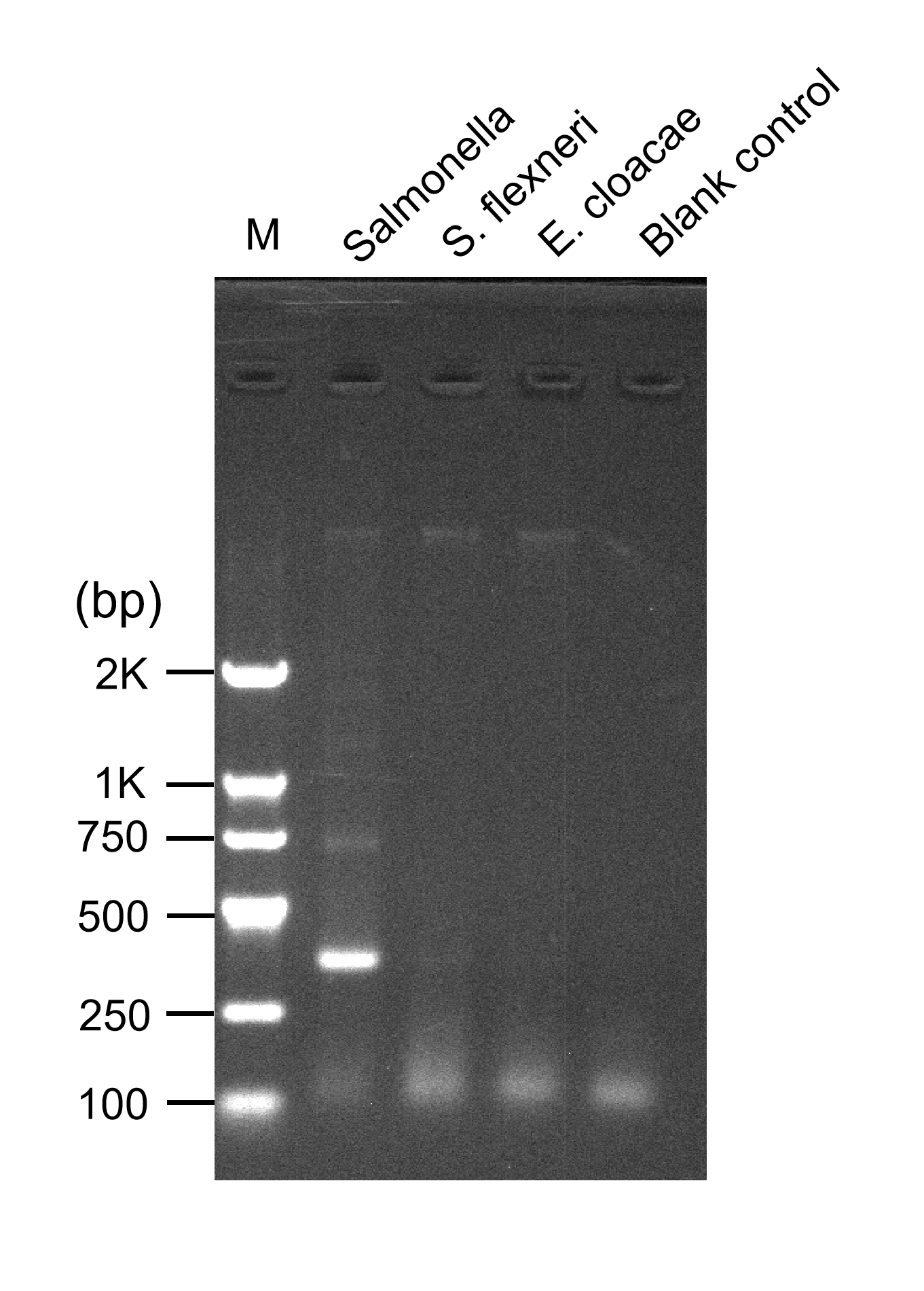


Fig S1 Agarose gel electrophoresis of RPA products for the specificity evaluation. M: DNA marker, Line 1: *Salmonella*, Line 2: *S. flexneri*, Line 3: *E. cloacae,* Line 4: blank control (ddH_2_O).
